# Associations between various kinds of parental support and physical activity among children and adolescents in Shanghai, China: gender and age differences

**DOI:** 10.1186/s12889-020-09254-8

**Published:** 2020-07-25

**Authors:** Jin-Tao Hong, Si-Tong Chen, Yan Tang, Zhen-Bo Cao, Jie Zhuang, Zheng Zhu, Peijie Chen, Yang Liu

**Affiliations:** 1 School of Physical Education and Sport Training, Shanghai University of Sport, Shanghai, 200438 China; 2 Institute for Health and Sport, Victoria University, Melbourne, 3001 Victoria Australia; 3 Shanghai Research Centre for Physical Fitness and Health of Children and Adolescents, Shanghai University of Sport, Shanghai, 200438 China; 4 School of Kinesiology, Shanghai University of Sport, Shanghai, 200438 China

**Keywords:** Children and adolescents, Chinese family, Exercise, Parental support, Physical activity, Shanghai

## Abstract

**Background:**

The associations between various kinds of parental support and children and adolescents’ physical activity (PA) are inconclusive. This study aimed 1) to examine the associations between various kinds of parental support and children and adolescents’ moderate-to-vigorous physical activity (MVPA), and 2) to examine gender and age differences in the association between each specific kind of parental support and MVPA.

**Methods:**

Using a multi-stage stratified and random cluster sampling method, 711 schools in Shanghai were selected (data were collected from October 2014 to February 2015). In total, 61,429 school-aged children (6–18 years old, 50.7% boys) and their parents were included. Self-reported questionnaires were used to measure sociodemographic characteristics, MVPA and various kinds of parental support, including parental encouragement (PAEN), parental involvement (PAIV), parental financial support (PAFS) and, parental modeling (PAMD). Descriptive statistics and Chi-square were used to report the level of MVPA and its difference across various kinds of parental support by gender groups. The logistic regression analysis was used to examine the associations between parental support and MVPA with odds ratios (ORs) and 95% confidence intervals (CIs).

**Results:**

Overall, 19.4% of children and adolescents accumulated MVPA at least 60 min/day. Boys were more physically active than girls (21.4% > 17.4%, *p* <  0.001). According to the observation of OR estimates, girls may be more susceptible to the influences from various kinds of parental support than boys (OR = 1.59, 1.61, 1.27 and 1.63, 95% CI: 1.19–1.73 among girls, and OR = 1.48, 1.60, 1.21, and 1.60, 95% CI: 1.14–1.69 among boys for PAEN, PAIV, PAFS and PAMD respectively). PAMD is the only one supportive kind that was positively associated with MVPA in both genders across all grades (OR = 1.29–2.98, 95% CI: 1.09–4.08 among boys; OR = 1.27–2.64, 95% CI: 1.10–4.10 among girls).

**Conclusions:**

Less than one fifth of children and adolescents accumulated 60 min MVPA per day. Various kinds of parental support have important effects on children and adolescents’ MVPA, which varied by gender and grades. PAMD, in particular, should be emphasized more than PAEN, PAIV and PAFS in family-based interventions aiming at increasing PA in the future.

## Introduction

Regular physical activity (PA) is related to numerous physical, psychological and social benefits for the growth and development of children and adolescents [1–3]. However, many children and adolescents do not engage in sufficient PA [4, 5]. In China, the most populous country in the world, only 13.1% of 9–17-year-old children and adolescents accumulated 60 min of moderate-to-vigorous physical activity (MVPA) per day, complying with the PA guidelines [6, 7]. Also, a decrease in PA levels from childhood to adolescence was observed among Chinese school-aged children [8]. Given the alarming epidemic of PA insufficiency, promoting PA among young people is a high public health priority [9].

PA of children and adolescents is influenced by many factors at multiple dimensions (e.g., individual, interpersonal, environment) [10]. Parents, at interpersonal level, play a crucial role in shaping children’s PA behaviour through various kinds of supportive behaviours [11, 12]. According to Beets and colleagues, social support from parents is defined as *“the functional characteristics associated with the interactions between a parent and his or her children in the context of intentionally participating in, prompting, discussing, and/or providing activity-related opportunities”* [12]. To be specific, parental support for PA includes a variety of operational ways, such as participating in PA jointly with children, providing logistic support (e.g. provision of transportation), acting as role models and encouraging children to do PA [12–15]. Positive associations between parental support and PA of children under 18 years old have been well documented in several systematic reviews and meta-analysis [16–18]. However, studies frequently investigated parental support categories aggregately as a combination, making it difficult to disentangle the specific relation of each kind of parental support on PA [12, 19]. Also, inconsistencies exist across the literature when it comes to findings on the association between one specific kind of parental support and PA. For example, many studies suggested that parental encouragement is positively associated with PA of children [14, 15, 20], whereas other research failed to confirm this [21]. The mixed findings can be found when exploring the associations between other types of parental support (e.g. providing financial support, being role models) and PA [14, 21, 22]. Therefore, the association between specific kind of parental support and PA of children and adolescents needs to be further investigated. Inspired by the study by Liu et al. [14], which examined the associations between different forms of parental support and PA among Chinese children and adolescents (*n* = 81,857), we choose to examine four kinds of parental support: parental encouragement (PAEN), parental involvement (PAIV), parental financial support (PAFS) and parental modeling (PAMD). By clarifying the specific role of a unique kind of parental support on children and adolescents’ PA, this study can help to determine whether any particular type is more significant than others. Furthermore, findings from our study may inform more effective family-based PA interventions aiming at increasing PA.

Gender and age may moderate the relationships between various kinds of parental support and children and adolescents’ PA [19, 23]. Although the associations were demonstrated to be similar in both genders [14, 15], the relative significance of each kind of parental support on PA may differ. Specifically, boys’ PA may be greatly promoted by tangible support (e.g. being transported to sports club), while girls’ PA may be largely influenced by intangible support (e.g. being encouraged by parents) [24]. Nevertheless, this is not always the case as one study revealed that emotional support from parents is conversely associated with PA among adolescent girls [25]. This may be owing to the feeling of being forced to be active when parents encouraged girls to play outside [26]. In addition, as age increases, the effects of social support from parents may become less important compared with supportive behaviours from other sources (e.g. peers) [19]. It has been documented that the influence from parents on PA of their children is stronger at a younger age (i.e. under 10 years old) and becomes weaker through adolescence [27, 28]. This may be explained by the reason that younger children are under less volitional control on their PA, while adolescents claim more autonomy over their PA behaviours [12, 17]. Thus, there is a necessity to further address these inconsistencies across the previous research.

Therefore, the purposes of this study are 1) to examine the associations between various kinds of parental support and MVPA among Chinese children and adolescents in Shanghai and 2) to examine gender and age differences in the association between each specific kind of parental support and children and adolescents’ MVPA.

## Methods

### Study design and sampling procedure

From October 2014 to February 2015, a large cross-sectional school survey organized by the Shanghai Municipal Education Commission was conducted in Shanghai, China. Using a multi-stage stratified and random cluster sampling method, a 2-stage cluster (non-probability) sampling was implemented as follows: 1) 711 primary, secondary and high schools from all 17 districts in Shanghai were selected; and 2) among these schools, 1–2 classes from each grade (Grades 1–12) were recruited. Each grade class was required to contain at least 20 students (10 boys and 10 girls). If the numbers of students did not meet as required, supplementary samples would be taken from other schools in the same district. Details about the study design can be found in elsewhere [29].

The study protocol and procedure were approved by the Institutional Review Board (IRB) of the Shanghai University of Sport (SUS). And the permission to carry out this study was received from the teachers and principals of those participant schools. The verbal consent was considered to be adequate and credible for conducting the survey according to the approval of IRB. All participations were not compulsory. Children and their parents were well informed, and verbal consents were obtained from all participants before the data collection.

### Participants

A total number of 78,516 school-aged children (Grades 1–12, 6–18 years old) and their parents were invited to fulfill this survey. 71,404 students (boys 51.0%) and 70,346 parents answered the self-reported questionnaires (response rates = 90.9 and 89.6%). It should be noticed that the term “parent” in this study refers to any guardian who is in charge of the health of the child (not only limited to biological parents). The reasons for students and parents not answering the questionnaires were: 1) a part of students could not answer the questionnaire because they were taking academic tests during data collection; 2) some students were absent due to physical diseases and they did not submit questionnaires afterward; 3) parents did not need to respond to self-reported questionnaires if their children did not answer questionnaires for any reason; and 4) parents could not answer their questionnaires because they were not available (i.e. not in Shanghai) during data collection and did not submit questionnaires afterward. The reasons for excluding some data were: 1) key variables were missing or in abnormal values (e.g. gender, grade); 2) the dependent variables were missing or in abnormal values (e.g. no answer for MVPA during the weekend); 3) independent variables were missing or in abnormal values (e.g. no answer for PAFS); and 4) the parent questionnaire and the student questionnaire were mismatched (e.g. identifications for match were missing, so we were not able to identify whose parents they are). After data cleaning, 61,429 eligible cases of school-aged children (boys 50.7%; mean age 11.77 ± 3.31 years old) and their parents were included in the analyses.

### Measurements of variables

In this study, MVPA and parental support were measured by an applied questionnaire designed for Chinese children and adolescents [29]. Those items have been confirmed as reliable and feasible measures for school-aged children in China [30], and they have been used in the study by Liu et al. [14]. Children and adolescents were required to report their demographic information, including gender (1 = male, 2 = female), age and grade (Grades 1 to 12). Considering the cognitive development, children below 11 years old completed self-reported questionnaires with the help of their parents or guardians.

#### MVPA

MVPA is defined as *“any kind of physical activity that increases your heart rate and make you breathe hard some of the time (including physical education time, exercising, sport training and various regular daily activities such as brisk walking, hiking and excursion)”* in the questionnaire. All students self-reported their physical activity by answering two items (children below 11 years old completed self-reported questionnaires with the help of their parents or guardians): 1) *“Over the past 7 days, how many days were you physically active for at least 60 minutes per day during weekdays (from Monday to Friday)?”* and 2) *“Over the past 7 days, how many days were you physically active for at least 60 minutes per day during weekend (from Saturday to Sunday)?”* The response options for the first question are *“0 = 0 day, 1 = 1 day, 2 = 2 days, 3 = 3 days, 4 = 4 days, 5 = 5 days”* and those for the second question are *“0 = 0 day, 1 = 1 day, 2 = 2 days”*. The participants who reported 7 days in total with at least daily 60 min of MVPA meet the PA guidelines [6]. A dichotomized variable with 1 = 7 days MVPA and 0 = other answers was computed.

#### Parental support

Parental supports were reported by parents or guardians of students. Various kinds of parental support were measured by four items: 1) *“Do you encourage your child to take part in physical activity/exercise?”*; 2) *“Do you join your child when he/she participates in physical activity/exercise?”*; 3) *“Do you provide financial support when your child participates in physical activity/exercise? (e.g. buy equipment, pay for sports training, etc.)?”*; 4) *“Do you serve as a role model for your child by engaging in physical activity/exercise?”*. Using a 5-point Likert-type scale, the response categories for each question are *“5 = very often, 4 = sometimes, 3 = not sure, 2 = not much, 1 = never”*. Questions 1 to 4 are successively considered as parental encouragement (PAEN), parental involvement (PAIV), parental financial support (PAFS) and parental modeling (PAMD) in this study. Parents who answered *“very often”* in each kind of parental support are considered as providing high support on children’s PA and others are considered as offering low support. A dichotomized variable with 1 = high support and 0 = low support was computed.

### Statistical analysis

All statistical analyses were conducted in SPSS version 22.0 software (SPSS Inc., Chicago, IL, USA). According to the purposes of this study, variables of gender, age (grade), MVPA, and parental support were included in the statistical analysis. Descriptive statistics and Chi-square were used to report the level of MVPA and its difference across various kinds of parental support by gender groups. The logistic regression analysis was used with MVPA as the dependent variable and each kind of parental support as independent variables to examine the associations between parental support and MVPA among children and adolescents by gender and grade groups with odds ratios (ORs) and 95% confidence intervals (CIs). All tests were considered statistically significant at an overall α level of 0.05 (two-sided).

## Results

The sample size and mean age of children and adolescents by gender are presented in Table 1. In total, 61,429 school-aged children (31,143 boys, 50.7%) were included, with a mean age of 11.77 years old (SD = 3.31).

**Table 1:** The sample size and age by gender

	Sample size		Age (years)	
	n	%	Mean	SD
Boys	31,143	50.7	11.78	3.39
Girls	30,286	49.3	11.76	3.22
Total	61,429	100.0	11.77	3.31

Table 2 shows the prevalence of meeting the PA guidelines and the proportion of each kind of parental support in low and high support groups by gender. Overall, 19.4% of children and adolescents met the PA guidelines. Boys were more physically active than girls (21.4% vs. 17.4%, *p* <  0.001). Regardless of gender, over 60% of parents provided high support of PAEN (boys: 62.5%, girls: 60.6%), while only around a quarter of parents provided high support of PAIV (boys: 25.5%, girls: 26.4%).

**Table 2:** The status of meeting the PA guidelines and various kinds of parental support

	Total		Boys		Girls	
	(*n* = 61,429)		(*n* = 31,143)		(*n* = 30,286)	
	n	%	n	%	n	%
MVPA	11,936	19.4	6660	21.4	5276	17.4*
Parental support						
PAEN						
Low	23,597	38.4	11,673	37.5	11,924	39.4
High	37,832	61.6	19,470	62.5	18,362	60.6
PAIV						
Low	45,470	74.0	23,192	74.5	22,278	73.6
High	15,959	26.0	7951	25.5	8008	26.4
PAFS						
Low	27,347	44.5	14,041	45.1	13,306	43.9
High	34,082	55.5	17,102	54.9	16,980	56.1
PAMD						
Low	38,791	63.1	19,721	63.3	19,070	63.0
High	22,638	36.9	11,422	36.7	11,216	37.0

*MVPA* Moderate-to-vigorous physical activity, *PA* Physical activity, *PAEN* Parental encouragement, *PAFS* Parental financial support, *PAIV* Parental involvement, *PAMD* Parental modeling

*Compared between boys and girls (*p* < 0.001)

Boys and girls who received high support of PAEN were more likely to meet the PA guidelines than their counterparts with low support of PAEN (boys: 23.8% vs. 17.4%, girls: 20.0% vs. 13.5%, both *p* <  0.001). Similar situations were observed in PAIV, PAFS and PAMD groups among boys and girls (Table 3).

**Table 3:** The prevalence of meeting the PA guidelines in low and high parental support groups

	Boys (*n* = 6660)		Girls (*n* = 5276)	
	n	%	n	%
PAEN				
Low	2034	17.4	1612	13.5
High	4626	23.8	3664	20.0
^a^*p*	< 0.001		< 0.001	
PAIV				
Low	4467	19.3	3450	15.5
High	2193	27.6	1826	22.8
^a^*p*	< 0.001		< 0.001	
PAFS				
Low	2759	19.6	2064	15.5
High	3901	22.8	3212	18.9
^a^*p*	< 0.001		< 0.001	
PAMD				
Low	3627	18.4	2809	14.7
High	3033	26.6	2467	22.0
^a^*p*	< 0.001		< 0.001	

*PA* Physical activity, *PAEN* Parental encouragement, *PAFS* Parental financial support, *PAIV* Parental involvement, *PAMD* Parental modeling

^a^Compared between the low and high parental support groups

Logistic regression analysis by gender revealed associations between various kinds of parental support and MVPA among boys and girls (Table 4). Compared with those who received low parental support, boys with high support of PAEN, PAIV, PAFS and PAMD had greater likelihoods of meeting the PA guidelines (OR = 1.21–1.60, 95% CI: 1.14–1.69). Similar findings were also observed among girls (OR = 1.27–1.63, 95% CI: 1.19–1.73).

**Table 4:** The associations between various kinds of parental support and children and adolescents’ MVPA

	Boys (*n* = 31,143)		Girls (*n* = 30,286)	
	OR	95% CI	OR	95% CI
PAEN				
Low	1		1	
High	1.48	1.39-1.57	1.59	1.49-1.70
PAIV				
Low	1		1	
High	1.60	1.51-1.69	1.61	1.51-1.72
PAFS				
Low	1		1	
High	1.21	1.14-1.28	1.27	1.19-1.35
PAMD				
Low	1		1	
High	1.60	1.52-1.69	1.63	1.54-1.73

*CI* Confidence interval, *MVPA* Moderate-to-vigorous physical activity, *OR* Odds ratio, *PAEN* Parental encouragement, *PAFS* Parental financial support, *PAIV* Parental involvement, *PAMD* Parental modeling; Reference category: the low support group of each kind of parental support

**Fig. 1:**
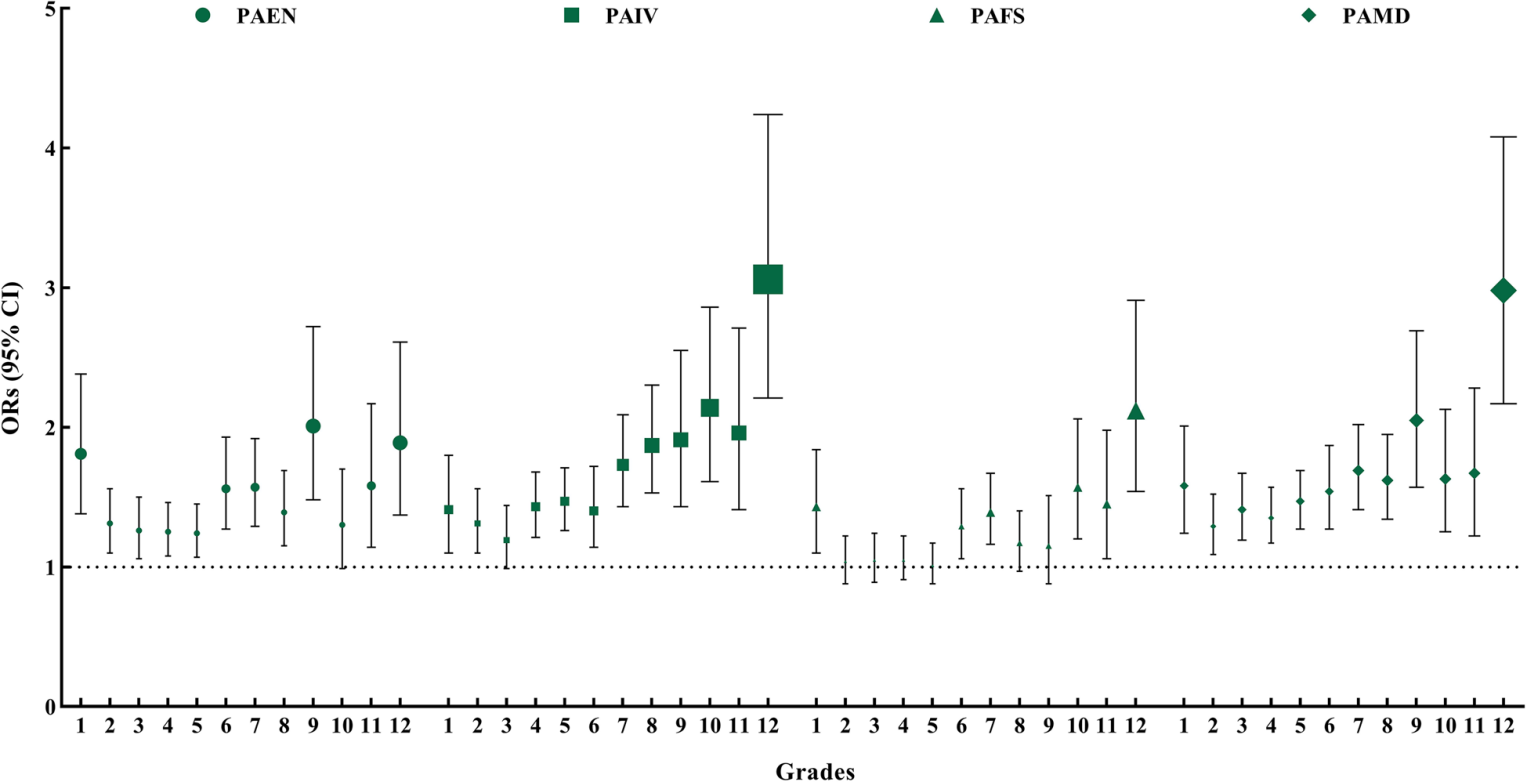
The associations between various kinds of parental support and boys’ MVPA by grades. *CI* Confidence interval, *PAEN* Parental encouragement, *PAFS* Parental financial support, *PAIV* Parental involvement, *PAMD* Parental modeling; Reference category: the low support group of each kind of parental support

**Fig. 2:**
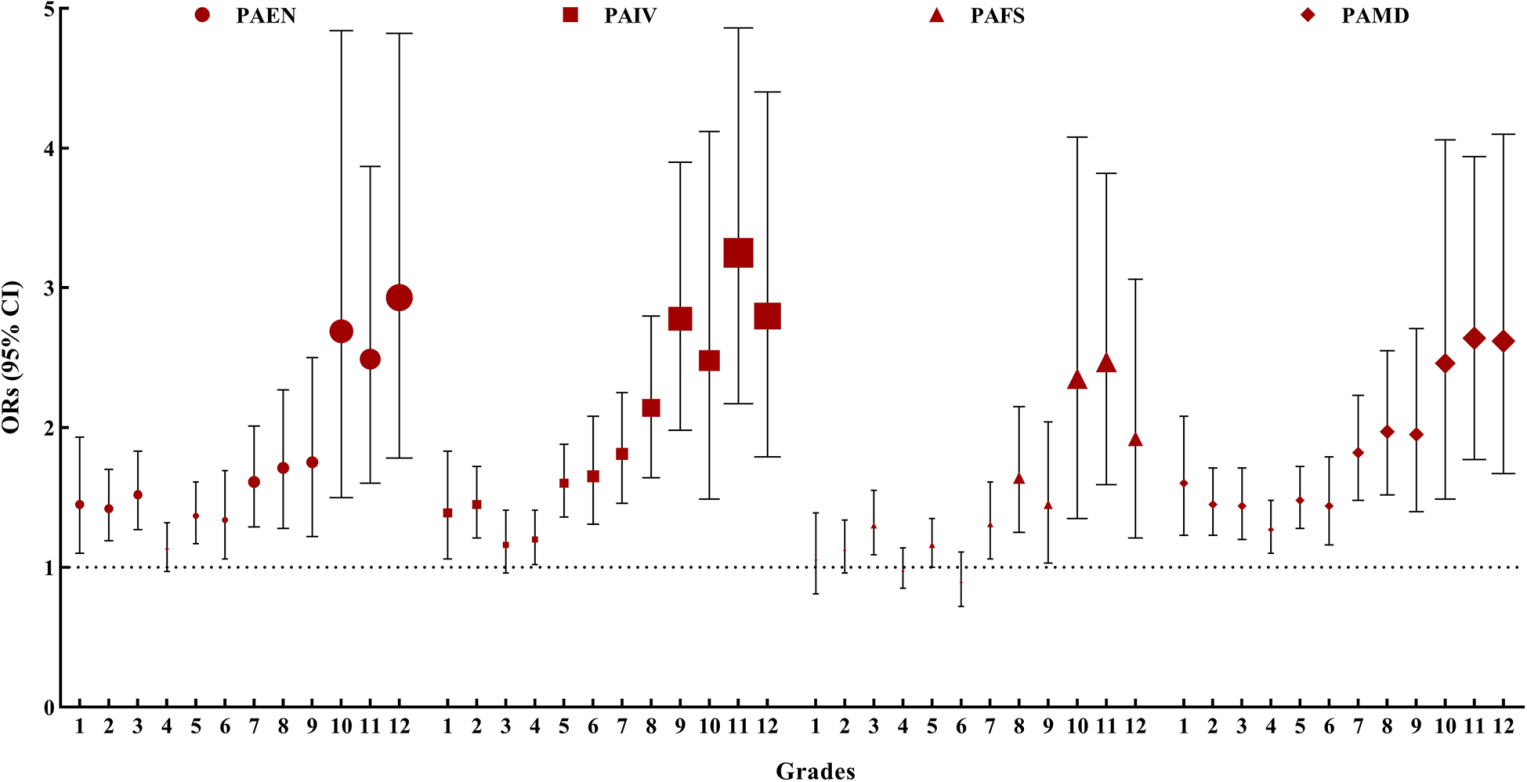
The associations between various kinds of parental support and girls’ MVPA by grades. *CI* Confidence interval, *PAEN* Parental encouragement, *PAFS* Parental financial support, *PAIV* Parental involvement, *PAMD* Parental modeling; Reference category: the low support group of each kind of parental support

Figure 1 and Fig. 2 illustrate the associations between various kinds of parental support and MVPA by grades among boys and girls. PAMD was the only kind of parental support that was positively associated with MVPA across all grades regardless of gender (OR = 1.29–2.98, 95% CI: 1.09–4.08 among boys; OR = 1.27–2.64, 95% CI: 1.10–4.10 among girls). PAIV was positively associated with boys’ and girls’ MVPA, except for MVPA of children in Grade 3 (OR = 1.31–3.06, 95% CI: 1.10–4.24 among boys; OR = 1.20–3.25, 95% CI: 1.02–4.86 among girls). PAEN was positive associated with boys’ and girls’ MVPA, except for MVPA of boys in Grade 10 and girls in Grade 4 (OR = 1.24–2.01, 95% CI: 1.06–2.72 among boys; OR = 1.34–2.93, 95% CI: 1.06–4.84 among girls). PAFS was positively associated with MVPA among boys in Grade 1, 6, 7 and 10 to 12 (OR = 1.29–2.12, 95% CI: 1.06–2.91), and among girls in Grade 3, 5 and 7 to 12 (OR = 1.16–2.47, 95% CI: 1.00–4.08).

## Discussion

Using a large representative sample of 61,429 students from Shanghai, China, our study showed that less than 20% of children and adolescents met the PA guidelines. Boys were more physically active than girls. In general, four kinds of parental support (PAEN, PAIV, PAFS, and PAMD) were all positively associated with children and adolescents’ MVPA in both genders. More significantly, the associations between each specific kind of parental support and young people’s MVPA differed by gender and grades. PAMD was found to be the only one kind of parental support that was positively associated with both boys’ and girls’ MVPA across all grades. PAIV and PAEN were also positively associated with boys’ and girls’ MVPA in most grades.

In accordance with many previous research findings [7, 14, 29, 31], we found low prevalence among children and adolescents of meeting the PA guidelines. Rapid economic development, urbanization, and dynamic social changes may explain the poor PA level among young people in Shanghai [32]. Align with prior findings [8, 14, 29], girls were less physically active than boys in the current study. Possible reasons for this include that boys had a higher level of MVPA during physical education class at school and they participated more in organized sport activities [29, 33].

In line with findings from other studies [14, 15, 20], we revealed that tangible and intangible parental supportive behaviours including PAEN, PAIV, PAFS, and PAMD were all positively associated with children and adolescents’ MVPA. One possible explanation is that strong social supports from parents facilitate children’s self-efficacy, which helps them to overcome the obstacles and then to do more PA [34, 35]. These findings, however, are contradicted with a study by Chiarlitti and Kolen [21]. Their study did not find significant relationships between parents’ PA, encouragement or financial support and children’ PA. The reason may be that the sample size of their study was too small (*n* = 30, 15 children and 15 parents) to statistically reveal the real relationships between different kinds of parental support and children’s PA. Although OR estimates in current study for boys and girls are not directly comparable because they are from different reference groups, the observation suggests that girls might be more influenced by parental supports than boys. In addition, the observations from OR estimates also indicate that PAMD may have more significant influences on girls’ MVPA than PAEN, PAIV and PAFS. However, it should be declared again that the OR estimates among girls of various kinds of parental support are not directly comparable since they are from different reference groups. These findings are also contradicted with studies by Mendonça and Farias [24] and Peterson et al. [25], which did not find significant relationships between PAEN or PAIV and PA among girls. One possible explanation is that participants in these studies were adolescents, who have more autonomy and control over PA behaviour than younger children. Adolescents’ PA may be less influenced by their parents, and may even be inhibited by some kind of parental support (e.g. forcing children to engaged in activities outside) [12, 17, 26]. Our confirmation on positive associations between various kinds of parental support and MVPA among children and adolescents adds more evidence to the literature, especially in China. Multi-dimensional social support comprising motivational, conditional and instrumental supports from parents should be integrated into family-based PA interventions [12].

Our further examinations on the associations between each kind of parental support and children and adolescents’ MVPA by gender and grades demonstrate that associations are not consistent among children and adolescents with different genders and grades. PAMD is identified to be the only supportive type that is positively associated with MVPA in both genders across all grades. It further supports the notion that parents play important role models in increasing children and adolescents’ PA level [13, 14, 22, 31]. For different ages, the relationships between PAMD and MVPA are stronger among adolescent boys in Grades 9 and 12, and adolescent girls in Grades 10 to 12. The resemblances exist in other three kinds of parental support – that is, stronger associations between PAEN, PAIV, and PAFS and children and adolescents’ MVPA are found among adolescent boys and girls, especially among girls in Grades 10 to 12. These findings confirmed that strong relationships still exist between parental support and adolescent PA with increasing age [17, 36]. Such relationship is particularly strong between PAIV and MVPA. This may be explained by that children and adolescents participating in PA with parents not only improve the self-efficacy to overcome difficulties but also increase the enjoyment of participation in PA [25, 34]. The successful and enjoyable experience may motivate them to do more PA. Consistent with Davison and Jago [37], PAFS is mostly positively associated with MVPA among adolescents for both boys and girls. Logistic support may play a more significant role as the age grows. It is interesting that stronger associations between various kinds of parental support and MVPA are all found among adolescents rather than children. Taken together, PAMD has lasting influence on children and adolescents’ MVPA across increasing age.

The first notable strength of this study is a large-sized sample from Shanghai, which benefits to further understand the PA prevalence among Chinese children and adolescents. In addition, to our knowledge, this study is one of few studies examining the association between various kinds of parental support and children and adolescents’ MVPA by gender and grades from 1 to 12. This study depicts relative differences of specific associations between PAEN, PAIV, PAFS, as well as PAMD and MVPA among boys and girls aged from 6 to 18 years old. However, several limitations should be mentioned. Firstly, it should be pointed out that this study used multi-stage sampling, and the basic unit is class or school, which may cause higher standard errors compared with a sampling of individuals. The data structure of the cluster may generate a larger confidence interval and therefore results in a misleading interpretation of the results, especially when the *p* value is near 0.05. Secondly, the PA level of children may be overestimated by using self-reported measures, especially among younger children. Thus, objective methods to measure PA among children and adolescents are needed in the future. Thirdly, in order to select eligible data, we removed about 14.0% of school-aged children and approximately 12.7% of parents samples. The drop-off of these samples has potential influences on findings. If these excluded sample data include a high percentage of children and adolescents with low level of PA and low parental support by parents, then the presented results may be overestimated. Last but not least, the results from this study only represent Shanghai, and it cannot be generalized to other areas in China. Also, the causal relationship is not able to be concluded due to the nature of the cross-sectional design. Future research should adopt an improved study design to further explore other areas of China with more diverse geographical characteristics.

## Conclusion

Our study showed that the percentage of children and adolescents meeting the PA guidelines was low, and boys were more physically active than girls. In general, various kinds of parental support were positively associated with children and adolescents’ MVPA. However, the associations between each specific kind of parental support and children and adolescents’ MVPA differed by gender across ages. In particular, parental role modeling should be emphasized more than parental encouragement, parental involvement and parental financial support in family-based interventions aiming at improving PA among children and adolescents.

## Data Availability

The datasets analysed in this study are available from the corresponding author on reasonable request.

## Abbreviations

CI: Confidence interval
IRB: Institutional Review Board
MVPA: Moderate-to-vigorous physical activity
OR: Odds ratio
PA: Physical activity
PAEN: Parental encouragement
PAFS: Parental financial support
PAIV: Parental involvement
PAMD: Parental modeling
SUS: Shanghai University of Sport
